# Retinoid Therapy for Neuroblastoma: Historical Overview, Regulatory Challenges, and Prospects

**DOI:** 10.3390/cancers16030544

**Published:** 2024-01-26

**Authors:** Atsushi Makimoto, Hiroyuki Fujisaki, Kimikazu Matsumoto, Yoshiyuki Takahashi, Yuko Cho, Yoshihiko Morikawa, Yuki Yuza, Tatsuro Tajiri, Tomoko Iehara

**Affiliations:** 1 Department of Laboratory Medicine, Tokyo Metropolitan Children’s Medical Center, Fuchu 183-8561, Japan; 2 Clinical Research Support Center, Tokyo Metropolitan Children’s Medical Center, Fuchu 183-8561, Japan; yoshihiko_morikawa@tmhp.jp; 3 Department of Hematology/Oncology, Tokyo Metropolitan Children’s Medical Center, Fuchu 183-8561, Japan; yuki_yuza@tmhp.jp; 4 Department of Pediatric Hematology/Oncology, Osaka City General Hospital, Osaka 534-0021, Japan; h-fujisaki@med.osakacity-hp.or.jp; 5 Children’s Cancer Center, National Center for Child Health and Development, Tokyo 157-8535, Japan; matsumoto-kmk@ncchd.go.jp; 6 Department of Pediatrics, Nagoya University Graduate School of Medicine, Nagoya 466-8560, Japan; ytakaha@med.nagoya-u.ac.jp; 7 Department of Pediatrics, Hokkaido University Hospital, Sapporo 060-8648, Japan; ymcho@med.hokudai.ac.jp; 8 Department of Pediatric Surgery, Faculty of Medical Sciences, Kyushu University, Fukuoka 812-8582, Japan; tajiri.tatsuro.909@m.kyushu-u.ac.jp; 9 Department of Pediatrics, Kyoto Prefectural University of Medicine, Kyoto 602-8566, Japan; iehara@koto.kpu-m.ac.jp

**Keywords:** neuroblastoma, high-risk, retinoids, retinoic acids, isotretinoin, retinoid therapy, tumor differentiation therapy, maintenance therapy, off-label issue

## Abstract

**Simple Summary:**

Tumor differentiation therapy using retinoids has a long history of clinical development and an established role as a part of the standard treatment for high-risk neuroblastoma. However, basic and clinical science related to this treatment is still immature in multiple respects. In the present review, we systematically expand on the basic and clinical science of retinoids for neuroblastoma treatment, off-label issues related to the treatment and possible countermeasures, and finally, prospects for this therapy.

**Abstract:**

Retinoids are vitamin A derivatives and include trans-retinoic acid, isotretinoin, tamibarotene, and bexarotene, all of which are currently available for clinical use. The clinical development of retinoid therapy for neuroblastoma has a history spanning more than four decades. The most promising agent is isotretinoin, which can contribute to improving event-free survival in patients with high-risk neuroblastoma by approximately 10% when administered over six months as maintenance therapy. Although isotretinoin is regarded as an essential component in the standard clinical management of high-risk neuroblastoma, its use for this purpose in the US and EU is off-label. To promote isotretinoin use in Japan as a treatment for neuroblastoma, our clinical research team is planning to launch an investigator-initiated, registration-directed clinical trial. The present review article discusses the basic science behind retinoid therapy, pre-clinical/clinical evidence on neuroblastoma, the concept of the proposed clinical trial, and prospects for this therapy.

## 1. Introduction

Neuroblastoma is a highly malignant, embryonal tumor originating in the neural crest which develops into the adrenal medulla and sympathetic nerves. The disease has a prevalence of approximately one per 7000 live births, and 800 cases are newly diagnosed annually in the United States [1]. The age-standardized incidence rate in children globally ranges from 4.1 to 15.8 per million population in individuals aged 0 to 14 years [2].

Neuroblastoma is also aggressive and capable of metastasizing to the lymph nodes, bone, and bone marrow. On the other hand, some less aggressive, early onset cases sometimes regress spontaneously or undergo tumor differentiation [3]. Among the numerous, prognostic variables are disease stage, age at diagnosis, tumor pathology, tumor cell ploidy, and *MYCN* amplification status, which can help distinguish the disease’s characteristics and predict the clinical outcome [4].

Although neuroblastoma is highly sensitive to cytotoxic chemotherapy, relapses frequently occur in high-risk patients. However, a recent treatment strategy consisting of multiagent chemotherapy, tumor excision, local radiation therapy, and mega-dose chemotherapy plus autologous stem cell transplantation (SCT) followed by anti-disialoganglioside (GD2) immunotherapy combined with tumor differentiation therapy using retinoids has achieved a five-year event-free survival rate of 56.6% [5,6]. Several basic as well as clinical studies [7,8,9,10,11,12,13,14,15,16] have culminated in the development of a tumor differentiation therapy using isotretinoin, a retinoid agent and an essential element of the multimodal therapy described above. The present review article discusses both the basic and clinical research into retinoids as neuroblastoma treatment, regulatory hurdles to the approval of isotretinoin as an anti-neuroblastoma agent in Japan, and the prospects of tumor differentiation therapy using retinoids.

A query of PubMed (National Library of Medicine) was performed in October 2023 using the search algorithm “retinoid” OR “retinoic acid” AND “neuroblastoma” without restrictions on date, article type, or language to screen the references in the present review. Two reviewers (A.M. and Y.M.) independently screened 1707 articles for relevance by title and abstract before screening the remaining articles through a full text review using the following inclusion criteria: (1) articles written in English; (2) articles mainly discussing the relationship between retinoids and neuroblastoma; (3) clinical and preclinical studies; and (4) availability of the full text. Studies enrolling the same patients as another study were carefully reviewed to determine their inclusion or exclusion.

## 2. The Basic Science of Retinoids

### 2.1. Discovery of Vitamin A and Its Utility as a Cancer Treatment

Vitamin A was found to be necessary for normal development in experiments involving rats more than 100 years ago [17]. The chemical structure of vitamin A and its precursor, β-carotene, was first described in 1931 by Karrer, who received a Nobel Prize in 1937 for his discovery [18,19]. Two types of vitamin A are found in the diet: retinyl esters present in animal products, such as eggs, meat, fish, and dairy foods, and pro-vitamin A (e.g., α-carotene, β-carotene, etc.), which is found in fruits and vegetables [17]. Retinoids or retinoic acids (RAs), the natural metabolites of vitamin A, are fat-soluble, unsaturated isoprenoids which have a low molecular weight and play an essential role in various aspects of mammalian physiology [17].

RAs in particular influence the growth, differentiation, and death of cells, and the dysregulation of retinoid signaling pathways causes tumorigenesis. For this reason, RAs have attracted attention as potential anticancer agents [17]. Clinical research on the cancer chemoprevention potential (in parallel with epidemiological studies investigating the effect of dietary vitamin A on human health) and anticancer potential of RAs gained impetus following the Food and Drug Administration (FDA)’s approval in 1982 of 13-cis retinoic acid (isotretinoin) as a treatment for severe, recalcitrant, nodular acne [20]. A series of clinical trials of the drug as a neuroblastoma treatment then ensued. The following chapters will lay out the basic as well as the clinical science underlying the development of isotretinoin as a treatment for neuroblastoma.

The ability of all-trans retinoic acid (ATRA) to induce differentiation in specific leukemia cells in both mice [21] and humans [22] prompted intensive research into potential clinical applications of the substance. Clinical trials of ATRA for acute promyelocytic leukemia (APL) achieved complete remission in newly diagnosed as well as recurrent cases [23,24]. Based on these findings, ATRA was approved as an APL treatment in 1995 by the FDA, the European Medicines Agency (EMA), and the Pharmaceuticals and Medical Device Agency (PMDA). To date, ATRA continues to play an integral role in the treatment of APL, including pediatric cases, in conjunction with other cytotoxic chemotherapies [25,26] and more recently with arsenic trioxide [27,28]. These combination therapies may enable the dosage and toxicity of ATRA to be reduced while increasing its efficacy by overcoming the resistance of tumor cells to the drug. The details of the clinical development of ATRA are outside the scope of this article, and readers are referred to literature which specifically discusses the drug’s development process.

Both isotretinoin and ATRA are naturally occurring, endogenous RAs which can be mutually interconverted by isomerase in the human body [17]. Because the physiological level of these RAs is far below that required to treat a malignancy, the administration of a clinical formulation is necessary to obtain a therapeutic effect [29]. However, the toxicity of the drugs, including muco-cutaneous dryness, hyperlipidemia, and hepatic dysfunction, are unavoidable when therapeutic dosages are administered. A number of synthetic RAs have been designed to reduce the toxicity as well as to increase efficacy. One synthetic RA, bexarotene, was approved by the FDA (1999), the EMA (2001), and the PMDA (2016) [30] for use against refractory cases of cutaneous T-cell lymphoma. Fenretinide [31,32,33,34] and tamibarotene [35], also synthetic RAs, are currently being evaluated for their potential as a treatment for neuroblastoma and will be discussed later in this article.

### 2.2. Retinoid Chemistry

**Figure 1:**
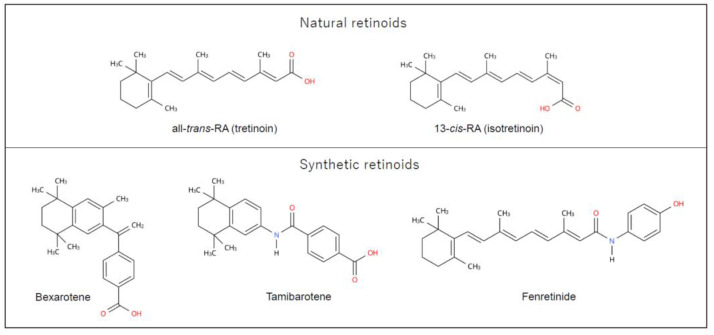
Chemical structure of retinoids used in clinical practice. A natural retinoid molecule consists of four isoprenoid units containing a hydrophobic part, the central polyene linker, and the polar region. Synthetic retinoids are generated by modifying the hydrophobic part and the central polyene linker to increase molecular stability. This figure was designed by authors using Ketcher 2.4.

Figure 1 shows the chemical structure of RAs. RAs are a class of compounds consisting of four isoprenoid units joined in a head-to-tail manner. They contain three regions: a hydrophobic part, the central polyene linker, and a polar region (usually characterized by a carboxylic acid) [17]. Synthetic RAs are primarily created by modifying the hydrophobic part and the central polyene linker to increase molecular stability. In many synthetic RAs, aromatic rings, called “arotinoids”, are often inserted into the central polyene linker. This modification results in low conformational flexibility, defining the length and directionality of the molecule, and producing an energetic benefit when the molecule binds to a retinoid receptor [17].

### 2.3. Retinoid Receptors and Their Signal Pathways

The anti-cancer mechanisms of RAs involve several gene signaling pathways. The most often cited signaling pathway is associated with two classes of receptors belonging to the nuclear hormone receptor superfamily [36]: the RA receptor (RAR) and the retinoid X receptor (RXR) [37]. This pathway is also considered to play an important role in neuroblastoma treatment [29,38]. When RAs bind to the ligand-binding domain (LBD) of RAR or RXR, conformational changes in the receptor form RAR-RXR heterodimers or RAR-RAR/RXR-RXR homodimers. These then interact with the RA-response elements (RAREs) located in target gene promoters to regulate gene expression in a retinoid-dependent manner [37,39]. Various polymorphisms have been identified in the promoter of many RA target genes involved in a wide variety of functions, including neuronal development and tumor suppression [40].

**Figure 2:**
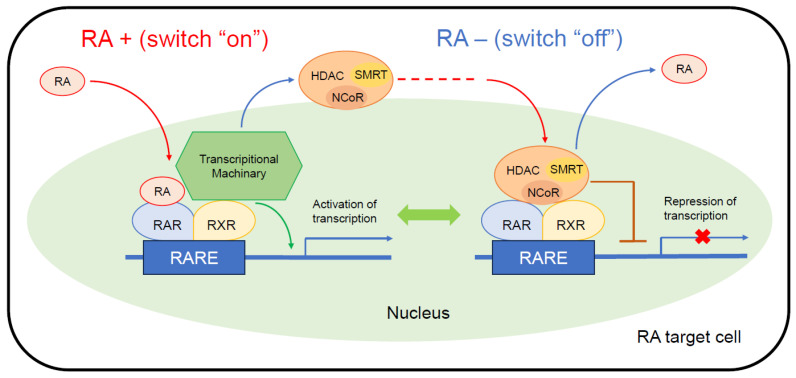
Coregulator exchange at RXR/RAR heterodimers. RARs function as modulators of transcription by recruiting coregulator complexes having HDAC activity. Retinoids function as a ligand activating or blocking gene transcription mediated via RXR/RAR heterodimers. This figure was designed by the authors. HDAC, histone deacetylase; NCoR, nuclear receptor corepressor complex; RA, retinoic acid; RARE, retinoic acid-response element; SMRT, silencing mediator of retinoic acid and thyroid hormone receptor.

In the absence of RAs as ligands (Figure 2, right), RAR-RXR heterodimers binding to DNA become associated with Co-repressor (CoR) complexes and block transcription. CoR complexes, such as nuclear receptor CoR (NCoR) and silencing mediator of retinoic acid and thyroid hormone receptor (SMRT), serve as adaptors recruiting high molecular weight complexes with histone deacetylase (HDAC) activity. These complexes deacetylate lysine residues in the N-terminal tail of histones and maintain chromatin in a condensed, repressed state over the target promoter [41].

When RA binds as a ligand to the RAR-RXR heterodimer (Figure 2, left), transcription is activated via the dissociation of the CoR complexes, which allows the recruitment of transcriptional machinery, consisting of the Mediator, RNA Pol II, general transcription factors (GTF), and nuclear excision repair (NER) factors, to the promoter.

There are three subtypes (α, β, and γ) of both RAR and RXR, each of which is coded for by separate genes. Although their individual, specific role in neuronal differentiation remains unclear, there is some evidence pointing to each RAR and RXR subtype having a distinct function. A previous study found that RARs were expressed in most neuroblastoma cell lines and in primary tumors [42]. While RARβ was only expressed in four of fourteen *MYCN*-amplified cell lines, it was able to be induced by ATRA in most of these cell lines [42]. No correlation was found between resistance to RA and the level of RAR or RXR expression. However, high RARβ expression was associated with good outcomes in patients with neuroblastoma, and overexpression of the gene by transfection appeared to increase the responsiveness of some neuroblastoma cell lines to RA [43]. Furthermore, an experiment involving siRNA-mediated silencing of RXRα and RXRβ found that the former was necessary for RA-induced neuronal differentiation of neuroblastoma-derived SH-SY5Y cells whereas the latter negatively regulated neuronal parameters related to neurite outgrowth and function [44].

Recent research has also uncovered extranuclear, nongenomic mechanisms, such as mitogen-activated protein kinase (MAPK) activation in the cytosol, which phosphorylates several molecules to regulate intranuclear gene expression [40]. Moreover, there are several other mechanisms of activity related to RAs, including the regulation of mitochondrial function [45], microRNAs [46], and *MYCN* signaling [47]. Both isotretinoin and ATRA are reportedly pan-RAR agonists, but several studies have claimed that isotretinoin has negligible affinity to RAR or RXR [48]. Therefore, these extranuclear, nongenomic mechanisms might play an important role in the therapeutic efficacy of isotretinoin for neuroblastoma.

Although all the details of isotretinoin activity against neuroblastoma are not known, using isotretinoin as maintenance therapy has become an essential facet of multimodal therapy for high-risk cases [7,8,9,10,11,12,13,14,15,16]. The following sections discuss the history of the preclinical and clinical development of RAs as a treatment for neuroblastoma.

## 3. Preclinical Evaluation of Retinoids for Neuroblastoma Treatment

As described above, neuroblastoma occasionally undergoes spontaneous regression and/or tumor cell differentiation to become a ganglioneuroma, which is a benign tumor. This discovery motivated research into the potential of cell differentiation as a treatment for neuroblastoma. The in vitro biological effects of isotretinoin and ATRA on neuroblastoma cell lines, including cell differentiation, sustained arrest of proliferation, and apoptosis, have been reported since the early 1980s [7,8,9,10,11,12]. The following is a summary of the preclinical, pharmacologic findings of isotretinoin and/or ATRA prior to the subsequent clinical studies.

Sidell et al., first demonstrated that ATRA induced concentration-dependent morphologic differentiation and growth inhibition in an LA-N-1 neuroblastoma cell line [7]. An ATRA dosage ranging from 10^−9^ to 10^−5^ M inhibited cellular proliferation in a concentration-dependent manner [7]. The growth curve of LA-N-1 cells showed complete inhibition from 72 to 96 h after exposure to ATRA, which continued for five days. Even after switching the medium to one without ATRA, this effect persisted at least for eight days, and the residual cell clusters displayed normal growth characteristics [7]. RA-induced, morphological differentiation, such as the formation of neurites, was significant at ATRA 10^−6^ to 10^−5^ M over 48 h and attained maximum differentiation after approximately four days. The cellular differentiation continued for seven to ten days even after the removal of ATRA from the medium [7].

A later study using seven neuroblastoma cell lines, including LA-N-2, LA-N-5, CHP134, SK-N-SH, KA, CHP100, and IMR32, confirmed these findings [8]. ATRA successfully inhibited cell growth and induced morphological differentiation in all but one (CHP100) cell line and induced neurite formation in four cell lines (LA-N-2, LA-N-5, CHP134, KA). On the other hand, IMR32 demonstrated cellular enlargement and vacuolization while SK-N-SH transformed into large, flat, epithelial-like cells [8]. The same study quantified and compared cellular retinoic acid-binding protein (CRABP) in LA-N-5 and CHP100. LA-N-5, the most RA-responsive cell line, contained approximately twice as much CRABP as CHP100, the least RA-responsive cell line [8]. Haussler et al. examined five cell lines (LA-N-1, IMR32, LA-N-5, SK-N-SH, CHP100), all of which contain CRABP, and found an association between CRABP and ATRA-induced inhibition of colony formation in soft agar [9].

Thiele et al., described a relationship between *MYCN* expression and ATRA-inducing neuronal differentiation in SMS-KCNR, another neuroblastoma cell line [10]. *MYCN* expression decreased within six hours of ATRA treatment and was followed by a decrease in the growth fraction (S + G2 + M) of cells at 48 h, then by morphological differentiation [10]. A recent study by Otsuka et al. supported a previous finding of accelerated MYCN protein degradation and neuronal differentiation in *MYCN*-amplified neuroblastoma cell IMR-32 following a combination treatment of peptide TNIIIA2 and ATRA [47].

Reynolds et al., compared the growth-inhibiting ability of ATRA and isotretinoin in 16 neuroblastoma cell-lines [12] and found the efficacy of ATRA 10 μM and isotretinoin 5 μM to be almost identical, with both achieving a growth inhibition as high as 1.7 logs [12]. Using 12 cell lines which were sensitized to RAs, another study found that a clinically achievable level of isotretinoin (5 μM) was significantly more effective than a clinically achievable level of ATRA (0.5 μM) in inhibiting cell growth [12]. An in vitro study assessing two courses of isotretinoin (5 μM) exposure for 14 days alternating with 14 days of rest found complete growth arrest in an *MYCN*-nonamplified SMS-LHN cell line for 120 days and an *MYCN*-amplified SMS-SAN cell line for up to 60 days [12].

## 4. Clinical Development of Isotretinoin for Neuroblastoma

Although both ATRA and isotretinoin were able to induce neuronal differentiation and growth arrest in neuroblastoma cell lines, isotretinoin was chosen for further clinical research in the US in part because it was better tolerated than ATRA by children [12,29,38]. The results of anecdotal clinical studies of isotretinoin for neuroblastoma, including a complete remission of bone marrow metastases and a two-year remission in one patient, provided corroboration for the choice [11]. Table 1 summarizes the history of the clinical development of isotretinoin for neuroblastoma, which will be discussed later in this chapter.

The first systematic, clinical evaluation of isotretinoin for neuroblastoma treatment was conducted by the Children Cancer Group (CCG), the results of which were reported by Finklestein et al. in 1992 [13]. Twenty-nine children aged less than 21 years with recurrent or progressive neuroblastoma following conventional therapy were enrolled. Oral isotretinoin was administered in a single, daily dose of 100 mg/m^2^. Two (9%) of twenty-two patients whose clinical response was evaluable demonstrated a positive response to isotretinoin therapy. The median overall survival was only 46 days. Safety evaluation was difficult due to the severity of the underlying neuroblastoma, which necessitated multiple supportive treatments, including blood transfusions. Approximately 50% of the patients were hospitalized for disease-related events for an average length of five days during the first course. The remaining patients experienced no toxicity. Adverse events, which were observed in two or more patients, included nine cases of cheilitis, five cases of fissured lip, three cases of xerosis, two cases of nausea/vomiting, three cases of abdominal pain, two cases of neutropenia <500/μL, four cases of thrombocytopenia <25,000/μL, and three cases of hemoglobin <8 g/dL. Most of these adverse events occurred during the first 28 days of therapy.

Owing to the disappointing results of the CCG study [13], isotretinoin was thought to be most effective in vivo against residual neuroblastoma remaining from maximal reduction of the tumor burden by mega-dose chemotherapy and SCT. At the same time, the intermittent administration schedule, which was based on an in vitro model published by Reynolds et al. [12], was considered to be ideal for higher dosages. Thus, a phase I clinical trial was conducted to determine the maximal tolerated dose (MTD), toxicity, and pharmacokinetics of isotretinoin in children with neuroblastoma following SCT [14]. Fifty-one eligible patients aged 2 to 12 years received oral isotretinoin in two equal doses daily over 14 days followed by a 14-day rest period for up to 12 courses. In total, 407 treatment courses with dose-escalation ranging from 100 to 200 mg/m^2^ were evaluated for toxicity. Dose-limiting toxicities, including hepatic dysfunction, hypercalcemia, skin rash, anemia, thrombocytopenia, and vomiting, occurred in six of nine patients at 200 mg/m^2^/day [14]. The MTD and recommended dosage were determined to be 160 mg/m^2^/day. All the toxicities resolved after the discontinuation of isotretinoin, and a complete response was observed in three cases of bone marrow metastasis [14].

These promising results led to a phase 3 clinical trial, CCG-3891, with a quasi-factorial design consisting of two sequential randomizations. The first randomization aimed to assess the superiority of high-dose chemotherapy with autologous SCT over three continuous courses of conventional chemotherapy in terms of event-free survival (EFS); the second part aimed to determine whether maintenance treatment with isotretinoin following cytotoxic chemotherapy could further improve EFS [15]. The isotretinoin maintenance therapy was administered in six courses at the same dosage as in the previous phase 1 trial, namely, oral isotretinoin 160 mg/m^2^/day in two equal doses daily for 14 days followed by a 14-day rest period. A comparison of the isotretinoin maintenance arm (n = 130) with a no-maintenance arm (n = 128) found three-year EFS rates of 46% and 29%, respectively (*p* = 0.027). Three-year overall survival (OS) did not differ significantly between the groups at 56% vs. 50%, respectively (*p* = 0.45).

The superiority in the survival rate of the isotretinoin maintenance arm was maintained in a follow-up study of the same patient cohort with an eight-year median follow-up. A trend in the improvement of five-year EFS (42 vs. 31%; *p* = 0.1219) and five-year OS (50 vs. 39%; *p* = 0.1946) was observed in patients randomly assigned to the isotretinoin maintenance arm, although the difference was non-significant [16]. A subgroup analysis in the same study found that the five-year EFS rate was 50% in patients randomly assigned to the autologous SCT and isotretinoin (n = 50) group but only 20% in patients assigned to the no-SCT and no-isotretinoin group (n = 53; *p* = 0.0038) [16]. Although five-year OS was not significantly higher in the patients with isotretinoin than in the patients without isotretinoin (50% vs. 39%; *p* = 0.1946), OS in the isotretinoin group was significantly higher on log(log(.)) transformation of the survival estimate at five years (*p* = 0.0006) [16].

Following the publication of the positive results of the CCG-3891 trial, the Children’s Oncology Group (COG) in the US declared isotretinoin maintenance therapy as the standard treatment for high-risk neuroblastoma, incorporating it into the standard therapeutic regimens in subsequent American clinical trials for high-risk neuroblastoma [5,49,50].

In contrast to the positive results of the CCG-3891 trial, another randomized trial of isotretinoin enrolling 175 patients from ten European countries failed to demonstrate the superiority of isotretinoin therapy. In this double-blind randomized trial, the participants received either isotretinoin 0.75 mg/kg/day or identical, placebo capsules for up to four years or until disease recurrence [51]. Three-year EFS in the isotretinoin arm (n = 88) and placebo arm (n = 87) was 37% and 42%, respectively (*p* = 0.62) [51]. Differences in the treatment effect among the various isotretinoin dosages and schedules suggested that achieving a pharmacologically effective drug level allowing a certain duration of exposure of neuroblastoma cells to the drug is necessary for isotretinoin to work as an anticancer agent. The following section will discuss the pharmacokinetic issues.

**Table 1:** Clinical development of isotretinoin for neuroblastoma.

Study Group	Study Design (Phase)	N	Dosage/Schedule	Target Condition	Findings	Ref.
CCG	Dose-finding (phase 1)	29	100 mg/m^2^/d QD	Refractory disease	Two patients had a clinical response. Median OS: 46 days	[13]
CHLA	Dose-finding (phase 1)	51	100–200 mg/m^2^/d BID for 14 d, 14 d rest	Patients after SCT	RD was 160 mg/m^2^/d. Three patients with BM met achieved CR.	[14]
CCG	Randomized, Open (phase 3)	258 *	160 mg/m^2^/d BID for 14 d, 14 d rest	Patients after SCT	Three-year EFS was significantly better in isotretinoin arm (46 vs. 29%).	[15,16]
EU	Randomized, Double-blind (phase 3)	175	0.75 mg/kg/d QD	Patients after SCT	Three-year EFS did not differ (37% in isotretinoin vs. 42% in placebo).	[51]

* Number of patients in second randomization. CCG, Children’s Cancer Group; CHLA, Children’s Hospital of Los Angels; EU, European Union; BID, twice daily; QD, once daily; BM, bone marrow; met, metastases; CR, complete response; d, day; SCT, stem cell transplantation; EFS, event-free survival; OS, overall survival.

## 5. Pharmacokinetic Issues and Countermeasures

For isotretinoin to exert its pharmacological effects in vivo, maintaining an adequate plasma concentration is necessary. As seen in the preclinical studies mentioned above, a plasma concentration ranging from 5 to 10 μM is required to maintain growth arrest and differentiation in neuroblastoma cell lines [11,12]. The pharmacokinetic details of a phase 1 study [14] demonstrated a linear increase in the mean peak serum level and area under the time-concentration curve (AUC) for a dosage range of 100 to 200 mg/m^2^ [52]. The peak serum concentrations were 4.9 ± 3.6 μM for 100 mg/m^2^ (n = 5), 7.2 ± 5.3 μM for 160 mg/m^2^ (n = 16), and 8.9 ± 10.0 μM for 200 mg/m^2^ [52]. A peak serum concentration exceeding 10 μM correlated with a high incidence (44%) of grade 3 to 4 toxicity [52]. The effectiveness of maintenance therapy in the CCG-3891 study, which was discussed in the previous section [15,16], probably resulted from the same type of active dosing, whereas the conservative dosing schedule in the European study [51] and the first CCG study [13] failed to demonstrate the therapeutic efficacy of isotretinoin.

Several factors influence the absorption, distribution, metabolism, and elimination (ADME) of isotretinoin. Isotretinoin has good permeability and poor solubility in the aqueous environment of the intestine, and its absorption is greatly enhanced by fatty foods. In contrast, the drug’s absorption and permeability are usually low during fasting. Veal et al. intensively investigated these factors in their pharmacokinetic studies [53,54] and found that swallowing intact capsules without extracting their contents was the biggest factor in achieving a high *C_max_* (4.0 ± 2.2 vs. 2.6 ± 1.8 μM) [54]. In this regard, the development of a drug formulation suitable for pediatric patients, such as a liquid or powder, may be desirable. However, the relatively small target population of neuroblastoma patients has stymied efforts to develop a new formulation, given the extremely high costs involved. Recently, the same group developed a novel, liquid formulation of isotretinoin and conducted a randomized, crossover clinical trial which demonstrated that the formulation had nearly identical pharmacokinetics, safety, and tolerability as the capsule formulation [55].

On the other hand, strategies for improving the bioavailability of isotretinoin, which are equally important for the treatment of neuroblastoma and acne vulgaris, have been implemented. Such strategies available include lidose technology [56] and more recently micronization technology [57], which utilizes a novel capsule formulation with a specific lipid vehicle to deliver the drug. Micronization technology in particular can substantially increase the surface area per unit mass of the drug and thereby increase its rate of dissolution and bioavailability [57]. Technologies such as these are likely to be crucial for achieving a higher and more stable *C_max_* by improving the drug’s bioavailability.

Two open-label, crossover studies compared micronized isotretinoin (Sun Pharmaceutical Industries, Inc., Cranbury, NJ, USA) 32 mg and its prototype, lidose isotretinoin (Absolica^TM^: Sun Pharmaceutical Industries, Inc., Cranbury, NJ, USA) 40 mg [57]. In one of these, a fed bioequivalence/food-effect study, 71 healthy, adult participants received a single dose of micronized isotretinoin 32 mg in a fed state, lidose isotretinoin 40 mg in a fed state, and micronized isotretinoin 32 mg in a fasted state. Bioavailability was assessed using the formula isotretinoin log-AUC_0–t_, log-AUC_0–∞_ and log-*C_max_* in blood samples taken pre-dosing and more than 96 h post-dosing. The 90% confidence interval for the baseline-adjusted geometric least squares mean ratios for log-AUC_0–t_, log-AUC_0–∞_, and log-*C_max_* fell within the 80–125% range for bioequivalence for micronized-isotretinoin 32 mg vs. lidose-isotretinoin 40 mg, both of which were administered in the fed state [57]. In the fasted state, 18 healthy, adult participants received a single dose of micronized-isotretinoin 32 mg and lidose-isotretinoin 40 mg. The results showed that micronized-isotretinoin 32 mg had approximately twice the bioavailability of lidose-isotretinoin 40 mg. Food had no effect on bioavailability and only a marginal effect on the extent of absorption of micronized isotretinoin 32 mg [57].

Another factor which might influence the pharmacokinetics of isotretinoin is the presence of biologically active metabolites. Of the major metabolites of isotretinoin, 4-oxo-isotretinoin is the most abundant [52,53,54,58]. In the phase 1 trial discussed above, the 4-oxo-isotretinoin level increased from day 1 to day 14 of isotretinoin administration in 64% of the patients [52]. A pharmacokinetic study conducted by Veal et al. found extensive accumulation of 4-oxo-13-cisRA during each course of treatment, with the plasma concentration of the metabolite being (4.677 ± 3.17 mM) higher than that of 13-cisRA (2.837 ± 1.44 mM) in 16 of 23 patients on day 14 of course 2 [53]. In another study by Veal et al., a statistically significant difference in the 4-oxo-isotretinoin *C_max_* value was observed on day 14 for CYP2C8*4 and CYP3A7*1C polymorphisms, although no clear impact of pharmacogenetics in determining the peak plasma concentration of isotretinoin itself was shown [54].

Sonawane et al. performed a series of experiments aimed at determining if 4-oxo-isotretinoin is an active metabolite of isotretinoin [58]. First, they compared the inhibitory effect of 4-oxo-isotretinoin with that of isotretinoin on six neuroblastoma cell lines (three with and three without *MYCN* amplification). Both 4-oxo-isotretinoin and isotretinoin demonstrated a similar inhibitory effect (*p* > 0.2 in all six cell lines) by inhibiting more than 90% of cell growth at the highest concentration in three cell lines (SMS-KANR, CHLA-20, and SMS-LHN). Second, they tested the inhibitory effect of 4-oxo-isotretinoin against both *MYCN* mRNA and protein in *MYCN*-amplified cell lines. The extent of the decrease relative to the control appeared to be identical for both 4-oxo-isotretinoin and isotretinoin. Third, they tested the ability of the substances to induce RARβ expression, which is reportedly associated with favorable clinical outcomes in neuroblastoma, as described in the previous section. Real-time reverse transcription PCR analysis demonstrated that both 4-oxo-isotretinoin and isotretinoin induced significantly higher (*p* < 0.05) RARβ mRNA levels than in vehicle control cells on day 10 in four *MYCN* gene-amplified and three *MYCN* non-amplified NB cell lines. No difference in the ability of the two substances to induce RARβ (*p* = 0.632) was observed. Finally, they confirmed neurite outgrowth and cell differentiation, which were promoted by both 4-oxo-isotretinoin and isotretinoin in SMS-KCNR and SMS-LHN cell lines.

Further investigation of the pharmacokinetics of isotretinoin and its metabolites in tandem with the development of a drug formulation suitable for childhood neuroblastoma is challenging but warranted as a step towards optimized tumor differentiation therapy using isotretinoin.

## 6. Pediatric Off-Label Use and Countermeasures

Although isotretinoin is an essential element of multimodal therapy for high-risk neuroblastoma, its use as a treatment for neuroblastoma is still off-label in most countries, including the US, EU, and Japan. This means that isotretinoin is not yet a part of the standard medical treatment from the regulatory point-of-view despite the scientific evidence for its efficacy having been established more than two decades ago. Our research group recently began a registration-directed, investigator-initiated clinical trial to test the safety and efficacy of micronized-isotretinoin as a step towards the approval of its use in Japan.

The Evaluation Committee on Unapproved or Off-labeled Drugs with High Medical Needs [59], established by the Ministry of Health, Labor and Welfare (MHLW) in 2009, recently concluded that isotretinoin qualifies as an unapproved drug with high medical need and therefore justifies urgent clinical development. Afterwards, discussions with PMDA, the regulatory agency in Japan, led to a tentative consensus that the accumulated data on the efficacy of isotretinoin for high-risk neuroblastoma in the US and EU may be extrapolatable to Japanese patients. This decision enabled planning for a relatively small clinical trial aimed at assessing the safety profile and pharmacokinetics of micronized isotretinoin (SPJ-101CA) for use as a treatment for high-risk neuroblastoma (Clinical Trial Registration: jRCT2031220687).

The inclusion criteria are (1) age 1 to 18 years; (2) a histopathological diagnosis of neuroblastoma or ganglioneuroblastoma; (3) high risk as defined by the International Neuroblastoma Risk Grouping system; (4) absence of progression after primary treatment, including chemotherapy, high-dose chemotherapy with hematopoietic stem cell transplantation, and radiotherapy; (5) 100 days or less after the most recent anticancer therapy; (6) absence of severe organ damage capable of interfering with the protocol treatment; (7) absence of an active, infectious disease; and (8) written informed consent from the patient and/or legal guardian. All the patients will receive oral SPJ-101-CA 128 mg/m^2^/day divided into two doses for 14 days followed by a 14-day break for six courses over a 28-day cycle. Concomitant treatment with chemotherapy will not be allowed. The primary endpoint is the severe adverse event rate with causality. The secondary endpoints include 1-year EFS, 1-year OS, the adverse event rate, and pharmacokinetics. Sixteen patients will be enrolled from March 2023 through August 2024. Follow-up observation will end in August 2025.

The clinical role of academic clinical trials in pediatric cancer drug approval has been actively discussed for decades [60]. The impact of regulatory approval of isotretinoin for neuroblastoma treatment will likely be significant in Japan, which is a member of the International Council for Harmonisation of Technical Requirements for Pharmaceuticals for Human Use (ICH). It is hoped that the planned study will result in advances enabling the development of effective treatment options for children with cancer, not least by providing a model of fair and efficacious collaboration among academia, industries, and regulatory agencies.

## 7. Prospects for Retinoid Therapy

As discussed above, isotretinoin is an established maintenance therapy for high-risk neuroblastoma. Current treatment regimens for high-risk neuroblastoma involve scheduling the administration of isotretinoin during the rest periods in anti-GD2 immunotherapy with the combination maintenance therapy lasting six months [5,6]. Although there is no clear evidence of synergy between isotretinoin and anti-GD2 immunotherapy, these drugs are concurrently administered to shorten the total treatment period. A long treatment duration for neuroblastoma is one of the biggest challenges to developing a new retinoid therapy because it is usually mildly cytotoxic and is indicated for use after consolidation therapy, including autologous SCT. A large-scale randomized trial is necessary to demonstrate the superiority of the new retinoid therapy over the current, standard isotretinoin regimen.

Because there are various other opportunities for using retinoids as neuroblastoma treatment besides maintenance therapy, it may also be applied in consolidation therapy or salvage induction therapy if the agent or regimen is sufficiently effective. There are three potential ways of developing a highly effective treatment, including using (1) an agent with strong cytotoxicity, such as fenretinide; (2) an agent/formulation with excellent bioavailability, such as micronized-isotretinoin; or (3) an agent with fine targetability, possibly via a novel drug delivery system, such as nanoparticles, to reduce adverse effects [61,62].

When developing a new tumor differentiation therapy, the use of retinoids should be evaluated as a single, independent treatment phase rather than as a part of maintenance therapy with anti-GD2 immunotherapy. If a new cell immunotherapy, such as chimeric antigen receptor (CAR), which expresses T cells targeting GD2, is established as a standard therapy to replace autologous SCT and anti-GD2 immunotherapy, the treatment period for neuroblastoma may be able to be shortened [63]. Such a treatment platform may be appropriate for testing a novel retinoid therapy as a new phase of treatment.

**Figure 3:**
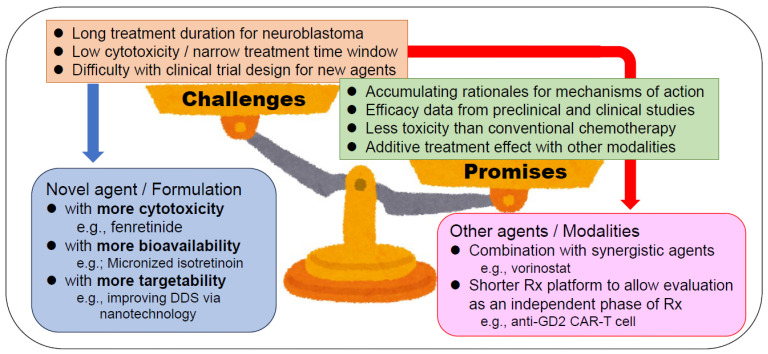
Promises, challenges, and countermeasures of a new tumor differentiation therapy based on retinoids. This figure was designed by the authors based on an illustration by Takashi Mifune with his permission. CAR-T cell, chimeric antigen receptor (CAR)-expressing T cells; DDS, drug delivery system; Rx, treatment.

Figure 3 summarizes the promises and challenges of a new tumor differentiation therapy based on retinoids.

Table 2 shows an overview of the clinical development of new retinoids for neuroblastoma. There are roughly two different directions of development.

One potential direction for further exploration involves the development of new retinoid agents with greater efficacy as a neuroblastoma treatment. Fenretinide, a synthetic retinoid which exerts a cytotoxic rather than maturational effect on neuroblastoma cell lines, has a long history of preclinical and clinical development [38]. Two phase 1 trials using a traditional capsule formulation of fenretinide have been completed [31,32]. Although the safety profiles were acceptable, oral administration of a very high-dose formulation involving a large number of capsules was needed to achieve an effective plasma concentration. A later phase 2 trial testing the recommended dosage of 2475 mg/m^2^ divided over three daily administrations in patients aged <18 years or 1800 mg/m^2^ divided into two daily administrations in patients aged 18 years or older every 21 days for a maximum of 30 courses achieved one partial response and 13 instances of prolonged stable disease in 59 evaluable patients [33]. To increase bioavailability, an oral, pulverized lipid complex (LXS) [34] and an intravenous formulation [64] are currently being developed.

Another synthetic retinoid, tamibarotene, functions as an RARα and RARβ agonist and was able to induce differentiation in SH-SY5Y and NH-12 cells to a greater extent than ATRA [65,66]. Recently, Nitani et al. reported the results of a phase 1 study of tamibarotene monotherapy targeting recurrent/refractory pediatric solid tumors in 22 patients (median age: 8 years) [35]. The subjects tolerated tamibarotene well, and no case of dose-limiting toxicity (DLT) occurred at any of the six dosages tested. However, none of the patients achieved a complete or partial response. The recommended dosage was determined to be 12 mg/m^2^/day for 21 days in a 28-day cycle [35]. Despite the huge effort involved in developing new retinoid therapies, no retinoid agent thus far has surpassed isotretinoin in terms of overall efficacy.

Combination therapy using RAs with other agents presents another potential area of research. As described in Section 2.3, RARs function as transcription modulators by recruiting coregulator complexes having HDAC activity. RAs work as ligands to activate or block gene transcription mediated by the RARs. This finding led to the idea of exploiting the synergy of combining an HDAC inhibitor with a natural RA in the treatment of neuroblastoma. Both in vitro and in vivo xenograft models have confirmed the synergy of retinoids and HDAC inhibitors. The combination of m-carboxycinnamic acid bis-hydroxamide (CBHA), an HDAC inhibitor, with ATRA led to synergistic cytotoxicity against neuroblastoma cell lines in in vitro and in vivo xenograft models [67,68]. Later studies using a combination of ATRA with the HDAC inhibitors, TSA, sodium butyrate, or vorinostat also demonstrated a synergistic effect inhibiting the growth of neuroblastoma cell lines in vitro [69]. Based on these preclinical findings, Pinto et al., conducted a phase 1 trial of vorinostat combined with isotretinoin for refractory/recurrent neuroblastoma [70]. The maximum intended dosage of vorinostat (430 mg/m^2^/day on days 1–4 and 8–11) combined with isotretinoin (160 mg/m^2^/day on days 1–14) was tolerable and led to more histone acetylation in surrogate tissues than at lower doses of vorinostat (*p* = 0.009). Although objective responses occurred, 17% of the evaluable patients achieved prolonged stable disease [70].

**Table 2:** Clinical development of other retinoids for neuroblastoma.

Drug	Study Design (Phase)	N	RD/Schedule	Efficacy Findings	Ref.
Fenretinide	Dose-finding (phase 1)	54	4000 mg/m^2^/d QD for 28 d, 7 d rest	Forty-one patients achieved SD for a median period of 23 months.	[31]
Fenretinide	Dose-finding (phase 1)	54	2475 mg/m^2^/d QD	One and thirteen of thirty patients achieved CR and SD, respectively.	[32]
Fenretinide	Safety/efficacy (phase 2)	62	2475 mg/m^2^/d TID (<18 y) 1800 mg/m^2^/d BID (≥18 y)	One and thirteen of fifty-nine patients achieved PR and SD, respectively.	[33]
Fenretinide LXS	Dose-finding (phase 1)	32	1500 mg/m^2^/d TID for 6 d, 15 d rest	Four and six patients with bone marrow disease achieved CR and SD, respectively.	[34]
Tamibarotene	Dose-finding (phase 1)	22	12 mg/m^2^/d BID for 21 d, 7 d rest	No patient achieved CR or PR.	[35]
Isotretinoin (I) + vorinostat (V)	Dose-finding (phase 1)	29	(I) 160 mg/m^2^/d BID for 14 d, 14 d rest (V) 430 mg/m^2^/d QD on d 1–4 and d 8–11	No patient showed an objective response. The RD achieved a sufficient vorinostat level to facilitate histone deacetylation in vitro.	[66]

BID, twice daily; TID, three times daily; QD, once daily; CR, complete response; PR, partial response; SD, stable disease; d, day; LXS, formulation of organized lipid complex; RD, recommended dosage.

There are several other possible combinations, such as isotretinoin plus vandetanib [71], fenretinide plus vorinostat [72] or lenalidomide [61], and tamibarotene plus 5-aza-2′-deoxycytidine [73], all of which have demonstrated a synergic, anti-neuroblastoma effect in vitro and in vivo. Basic research aiming to find new retinoid derivatives and synergistic combinations with various other agents is ongoing and will hopefully accelerate the clinical development of retinoid therapy for neuroblastoma [74]. Considering that most of the treatment phases for high-risk neuroblastoma are quite toxic for children, reducing the need for toxic tumor differentiation therapy can expand the application of such regimens not only as maintenance therapy following mega-dose chemotherapy and SCT but also as salvage treatment for heavily treated relapsed or refractory patients with neuroblastoma.

## 8. Conclusions

State-of-the-art multidisciplinary treatment consisting of multiagent chemotherapy, tumor excision, local radiation therapy, and mega-dose chemotherapy with SCT followed by anti-GD2 immunotherapy with tumor differentiation therapy using retinoids has dramatically improved the survival of patients with high-risk neuroblastoma. However, more than 40% of patients still experience a relapse which later becomes terminal. Therefore, further efforts to improve the treatment efficacy of these regimens are strongly warranted. Although the history of tumor differentiation therapy is long, there is still plenty of room for improvement either through the development of new, synthetic agents or of new combination therapies. Rapid progress in genetics and molecular science will hopefully provide novel and promising avenues for breakthrough research in tumor differentiation therapy.

## Data Availability

Data are contained within the article.
